# Effects of sumac fruit and prickly ash leaf extract on the intrinsic technological, sensory and safety quality properties of chicken fillets

**DOI:** 10.1016/j.fochx.2026.104691

**Published:** 2026-10-26

**Authors:** Sushmita Moirangthem, Gopal Patra, Subhasish Biswas, Laura Alessandroni, Santanu Nath, Arun K. Verma, Pramod Kumar Nanda, Arun K. Das, Mohammed Gagaoua

**Affiliations:** a Department of Livestock Products Technology, West Bengal University of Animal and Fishery Sciences, Kolkata 700 037, India; b School of Pharmacy, Chemistry Interdisciplinary Project (CHIP), University of Camerino, Via Madonna delle Carceri, 62032 Camerino, Italy; c Eastern Regional Station, ICAR-Indian Veterinary Research Institute, 37 Belgachia Road, Kolkata 700 037, India; d Goat Products Technology Laboratory, ICAR-CIRG, Makhdoom, Mathura-281 122, India; e PEGASE, INRAE, Institut Agro, 35590 Saint-Gilles, France

**Keywords:** Chicken fillets, Quality properties, Sumac fruits, Prickly ash leaves, Principal component analysis

## Abstract

This study evaluated the preservation efficacy of 10% (*w*/*v*) dip solution of sumac (*Rhus chinensis* Mill.) fruit (SF) and prickly ash (*Zanthoxylum acanthopodium* DC.) leaf (PAL) and assessed their effects on the quality of chicken fillets during 9 days of refrigerated storage (4 ± 1 °C), compared with untreated control. PAL extract had higher total phenolics (148.9 μg GAE/mg), while SF extract was richer in flavonoids (62.4 μg GAE/mg) and showed stronger DPPH and ABTS radical-scavenging activity. Multiple intrinsic quality attributes like pH, water-holding capacity, thiobarbituric acid reactive substances (TBARS), color, microbiological safety, and sensory properties were monitored. Both of the extracts (SF and PAL) showed distinct preservation profiles linked to their phytochemical and antioxidant properties. SF extract exhibited stronger radical-scavenging activity coupled with better controlled lipid oxidation and microbial growth, while PAL extract yielded comparatively more favorable sensory responses. Multivariate analysis further showed that SF and PAL treated groups were clearly separated from the untreated control through coordinated changes in oxidative, microbiological, technological, and sensory traits, highlighting their preservation efficacy. The findings indicate that variable preservation efficacy of SF and PAL extracts is driven by their intrinsic chemical characteristics and interactions within the complex chicken-muscle matrix, thereby providing a mechanistic basis that supports their practical application as natural preservatives for fresh meat.

## Highlights

• Sumac fruit and prickly ash leaf extracts improved the shelf life of chicken fillets
• Due to its high flavonoid content, sumac reduced lipid oxidation by 58%.
• Treated fillets were safely preserved 4 days longer than untreated (control)
• Sumac suppressed diverse pathogens, keeping microbial levels below spoilage limits for 9 days
• Prickly ash leaf extract-maintained the color of chicken fillets through lignans
• Multidimensional analyses showed sumac improved chicken fillet quality over time.

## 1 Introduction

Plant-derived materials including leaves, flowers, fruits, roots, peels, and barks are increasingly being investigated as sources of natural compounds with antioxidant and antimicrobial properties (Elgadir et al., 2025). These materials mostly contain diverse phenolic compounds, such as phenolic acids, flavonoids, phenolic diterpenes, and volatile compounds. Depending on their chemical composition, such constituents can retard lipid oxidation through radical-scavenging and metal-chelating activities, while also inhibiting microbial growth by disrupting cell membranes, or interfering with essential cellular processes. These properties have encouraged the use of plant extracts as potential clean-label ingredients for preserving a range of foods, including comminuted and whole-muscle meat products (Das et al., 2024; Shehata et al., 2026). Nevertheless, the effectiveness of a plant extract cannot be inferred from its in vitro antioxidant or antimicrobial activity alone, because its performance may change considerably after incorporation into a complex food matrix.

Chicken meat is valued for its high-quality protein, relatively low-fat content in lean cuts, and favorable fatty-acid profile. However, the presence of polyunsaturated fatty acids also makes chicken meat susceptible to oxidative deterioration during refrigerated storage conditions. Lipid oxidation may result in the formation of undesirable off-flavors and may contribute to changes in color, flavor, and other quality attributes (Connolly & Campbell, 2023). Likewise, the high-water activity, nutrient-rich composition, and suitable pH of raw chicken create favorable conditions for the growth of spoilage and pathogenic microorganisms. As a result, chilled chicken meat is prone to rapid quality deterioration, leading to a reduced marketable shelf life and heightened microbiological risks. Such changes not only diminish consumer acceptance but also cause huge economic losses across the poultry supply chain (Alessandroni et al., 2024; Das et al., 2019). These challenges have spurred growing interest in natural preservation systems with an aim at reducing the dependence on conventional additives. However, the replacement of synthetic preservatives should be guided by demonstrated antioxidant and antimicrobial efficacy and sensory acceptability instead of assuming that plant-derived ingredients are inherently safer.

Plant extracts and essential oils have, therefore been investigated as alternatives or complementary preservation agents, because many contain phenolic compounds and other constituents capable of simultaneously limiting oxidation and inhibiting microbial growth (Lemoni et al., 2025; Stabnikova et al., 2026). Once incorporated into a complex muscle food matrix, the extract constituents interact with muscle proteins, lipids, pigments and endogenous water-soluble components. However, their real-world practical performance does not always depend on in-vitro antioxidant or antimicrobial assays alone. Their efficacy in a complex food system is influenced by many factors, including plant part used, cultivar, extract chemistry, solvent and concentration, application method, packaging, storage conditions etc. (Aguiar Campolina et al., 2023; Hyldgaard et al., 2012).

Among the less extensively studied botanical resources, *Rhus chinensis* Mill. and *Zanthoxylum acanthopodium* DC. offer promise as candidates for meat preservation. The fruit of *R. chinensis*, commonly known as Chinese sumac, contains phenolic acids, tannins, flavonoids, and other phenolic constituents that have been associated with antioxidant and antimicrobial activities (Zhang, Zhang, et al., 2022). *Z. acanthopodium*, commonly referred to as andaliman and related to the prickly ash group, is an aromatic plant traditionally used as a spice and medicinal resource in parts of Asia (Yang et al., 2022). Its leaves and other tissues contain variable proportions of volatile constituents, terpenoids, flavonoids, alkaloids, and phenolic compounds, and extracts of the species have shown antioxidant or antimicrobial potential in laboratory studies (Adrian et al., 2023; Misra et al., 2013). Previous research has demonstrated the potential of films containing *Z. rhetsa* seed-cover extract for extending the refrigerated shelf life of ground chicken meat (Muppalla & Chawla, 2018). Likewise, incorporation of Sicilian sumac (*Rhus coriaria* L.) into beef burgers has exhibited antioxidant properties with reduced lipid peroxidation, while influencing color and sensory characteristics (Grassi et al., 2024).

Literature search indicate the use of extract, or a film-based delivery system of different species of sumac (e.g., *Rhus coriaria* L., *R. typhina*, *R. aromatica*, *R. copallinum*, *R. javanica* L., *R. tripartita*) and prickly ash (e.g., *Zanthoxylum zanthoxyloides*, *Z. clava-herculis* L., *Z. gilletii*, *Z. chalybeum*, *Z. martinicense* (Lam.) DC., *Z. limonella*, *Z. armatum* DC, *Z. bungeanum* Maxim., Rutaceae) plant derived material by different researchers against various ailments, and food-preservation (Adrian et al., 2023; Alsamri et al., 2021; Bursal & Köksal, 2011; Grassi et al., 2024; Misra et al., 2013; Muppalla & Chawla, 2018; Uçuk & Spence, 2026; Yang et al., 2022). However, evidence remains limited on the direct application of *R. chinensis* fruit and *Z. acanthopodium* leaf extracts to raw chicken fillets. It is well documented that the phytochemical profile of the same plant parts can also differ substantially affecting their biological activities, as influenced by many factors such as cultivars/ species, stage of maturity, geographical conditions, extraction procedures employed etc. (Li et al., 2022). Therefore, the present study was undertaken to compare the preservation effects of *R. chinensis* fruit extract and *Z. acanthopodium* leaf extract applied directly to raw chicken fillets and evaluated their efficacy under identical refrigerated storage conditions. Instead of assuming that all plant phenolics behave similarly, the study examines extract-specific responses by integrating antioxidant and antimicrobial activity with physicochemical, oxidative, sensory, and microbiological quality attributes.

## 2 Materials and methods

### 2.1 Plant parts, chemicals and chicken fillets

Sumac (*Rhus chinensis* Mill) fruits and prickly ash (*Zanthoxylum acanthopodium* DC) leaves were obtained from the local market of Imphal, Manipur, India and authenticated by a botanist. Various chemicals, and reagents such as trichloroacetic acid (TCA), 2-thiobarbituric acid, 1, 1diphenyl 2-picrylhydrazyl (DPPH), 2,2′-azinobis (3-ethylbenzothiazoline-6-sulonic acid) (ABTS), quercetin, Folin–Ciocalteu's reagent, sodium carbonate, gallic acid, sodium nitrite, sodium hydroxide etc. were purchased from standard firms like Sigma-Aldrich, USA and SRL, Mumbai, India. Media for antimicrobial studies were purchased from Hi-Media, Mumbai, India and SRL, Mumbai, India. Live poultry birds (*Gallus gallus*, Linnaeus) were purchased from the local market (Belgachia) Kolkata. The birds were slaughtered and prepared in the poultry processing unit of the Department as per standard method (Das et al., 2004). After hygienic dressing, skinless whole chicken breast muscle was separated and deboned to obtain fillets.

### 2.2 Preparation of plant extracts

The plant materials (sumac fruits and prickly ash leaves) were cleaned, visually inspected and dried under natural sunlight prior to grinding. The dried plant materials were grounded using mixer grinder to get fine powder and then sieved. The use of natural drying represents a practical preparation approach; however, variation in drying conditions may influence the recovery and stability of plant bioactive constituents and is, therefore recognized as a limitation of the present study. Individual plant powder (4 g) was extracted with 36 mL of aqueous-ethanolic solution (H_2_O: EtOH 60: 40, *v/v*). After constantly stirring for 3 h in a BOD shaker, the content was kept in a water bath (60 °C) for 1 h. After centrifugation at 4000 rpm for 15 min, it was filtered. The filtrate was then concentrated using a rotary evaporator (LABOAO, Model RE52A, China) under reduced pressure at 45 °C. Finally, the residual fraction was dissolved in sterile distilled water to prepare different concentrations of the extract for further in-vitro studies.

### 2.3 Bacterial strains

The antimicrobial activities of the sumac fruit (SF) and prickly ash leaf (PAL) extracts were tested against four bacterial strains viz. *Staphylococcus aureus* (ATCC-25923), *Listeria monocytogenes* (ATCC-19111), *Escherichia coli* (ATCC-25922) and *Salmonella Typhimurium* (ATCC-14028).

### 2.4 Analysis of plant extracts

#### 2.4.1 Tentative GC–MS based profiling of volatile and GC-amenable constituents

Plant powders were characterized by gas chromatography–mass spectrometry (GC–MS) using a triple quadrupole instrument (GCMS-TQ8030, Shimadzu, Japan) for profiling GC-amenable volatile and semi-volatile constituents in the plant materials. Briefly, chromatographic separation was carried out on an Rxi-5 ms capillary column (30 m × 0.25 mm, Restek), and the mass spectrometer was operated in Q3 scan mode over an *m*/*z* window of 40–700 at a scan rate of 2500 scans/s. The electron ionization (EI) was performed at 70 eV. The total run time ranged from 3 to 71 min, allowing elution and detection of both early- and late-retaining components. Individual components were identified by comparison with entries in the National Institute of Standards and Technology (NIST) mass spectral library, giving tentative identification of the major phytochemicals present in the plant powders. Because many plant polyphenols are non-volatile and thermally labile, the present GC–MS procedure should not be regarded as a comprehensive characterization of the native phenolic profile. Accordingly, the GC–MS results are presented as a qualitative screening of detectable constituents rather than as definitive quantitative identification of all phenolic compounds.

#### 2.4.2 DPPH radical scavenging activity

The free radical scavenging activity (RSA) of SF and PAL extracts was assessed using the DPPH assay following the procedure of (Rahman et al., 2015), with minor modifications. Briefly, increasing volumes (0.2, 0.4, 0.6, 0.8, and 1.0 mL) of plant extract solution (1 mg/mL) were transferred into separate test tubes and each volume was adjusted to 4 mL with distilled water. Subsequently, 1 mL of a 1 mM DPPH solution prepared in methanol was added to each tube. The mixtures were vortexed and then allowed to stand in the dark at room temperature for 30 min to permit the reaction between the antioxidant compounds and DPPH radicals. A control was prepared by mixing 1 mL of the DPPH solution with 4 mL of distilled water under the same conditions. After incubation, the decrease in absorbance was recorded at 517 nm using a UV–Vis spectrophotometer (Eppendorf BioSpectrometer, USA), and the radical scavenging activity (RSA) was calculated from the difference between sample and control readings.

The RSA was measured using the following formula:

$$\mathrm{RSA}\left(\%\right)=\frac{\mathrm{Ac}-\mathrm{As}}{\mathrm{Ac}}x\;100$$

Where, Ac is the absorbance of the control sample, and As the absorbance of the extract. The IC_50_ values were calculated from scavenging percentage of radical formation in presence of a test substance.

#### 2.4.3 ABTS radical scavenging activity

The ability of the extracts to neutralize ABTS radicals was evaluated using the decolourization assay (Re et al., 1999), with minor adjustments. First, a 7 mM stock solution of ABTS was prepared in distilled water. This solution was then mixed with potassium persulfate to a final concentration of 2.45 mM and kept for about 16 h in dark at room temperature to generate the ABTS- + radical cation, that gives a characteristic blue–green color.  Before use, the ABTS- + stock was diluted with methanol until its absorbance at 734 nm reached approximately 0.85 ± 0.05, providing a working solution of suitable optical density. An aliquot of 3 mL of this standardized ABTS- + solution was then combined with 1 mL of the extract at different concentrations and incubated for 4 min at room temperature. After incubation, the decrease in absorbance at 734 nm was recorded with a spectrophotometer, and the percentage scavenging activity was calculated relative to the control containing ABTS- + without extract. The percentage of inhibition was calculated using the formula:

$$\mathrm{Inhibition}\;\left(\%\right)=\frac{A0-A1}{A0}x\;100$$

where, A_0_ is the absorbance of control and A_1_ is the absorbance of sample. IC_50_ value was also determined using a standard curve.

#### 2.4.4 Total phenolics content

The total phenolics content (TPC) of the plant extracts was quantified using the Folin–Ciocalteu colorimetric assay (Sánchez-Rangel et al., 2013), with minor changes. In brief, 100 μL aliquots of the plant extract at different dilutions were transferred into test tubes, followed by the addition of 0.75 mL of Folin–Ciocalteu reagent. The volume in each tube was then brought up ten-fold with distilled water and the mixture was allowed to stand for 5 min at room temperature. Subsequently, 750 μL of a 7.5% (*w*/*v*) sodium carbonate solution was added to each tube, and the reaction mixtures were incubated in the dark for 90 min at ambient temperature to allow full color development. The absorbance of the resulting blue complexes was recorded at 725 nm against an appropriate reagent blank. The TPC values were obtained from a calibration curve constructed with standard gallic acid solutions and expressed as milligrams of gallic acid equivalents (GAE) per gram of extract.

#### 2.4.5 Total flavonoid content

The total flavonoid content of the extracts was measured using the aluminum chloride colorimetric assay (Wu et al., 2021), with minor changes. Serial dilutions of each extract were first prepared, and 1 mL from each dilution was transferred into a test tube containing 4 mL of distilled water. To this mixture, 0.3 mL of 5% sodium nitrite solution was added, followed by 0.3 mL of 10% aluminum chloride solution. After allowing the reaction to proceed for 6 min at room temperature, 2 mL of 1 M sodium hydroxide was added, and the volume was adjusted to 10 mL with distilled water. The resulting-colored solution was mixed well, and its absorbance was recorded at 510 nm against a reagent blank. Catechin served as the reference standard, and a calibration curve was constructed. The flavonoid content in the samples was expressed as milligrams of catechin equivalents per gram of extract (mg CE/g), based on the corresponding regression equation.

### 2.5 Antimicrobial activity of plant extracts

#### 2.5.1 Agar well diffusion assay

The antibacterial activity of the extracts was evaluated by the agar well diffusion technique (Singh et al., 2009). Test bacterial suspensions were first adjusted to the 0.5 McFarland standard, corresponding to approximately 1 × 10^8^ CFU/mL, and uniformly spread over Mueller–Hinton agar plates using sterile cotton swabs.  Wells of 6 mm diameter were aseptically punched into the agar with a sterile cork borer, and their bases were sealed with 50 μL of sterile molten nutrient agar. Each well was then filled with 100 μL of the extract at the desired concentrations, and the plates were incubated at 37 °C for 24 h. Antibacterial efficacy was assessed by measuring the diameter of the clear zones of inhibition (mm) surrounding the wells against each test organism.

#### 2.5.2 Estimation of minimum inhibitory concentration (MIC)

The minimum inhibitory concentration (MIC) of the plant extracts was determined using a resazurin-based broth microdilution assay (Sfeir et al., 2013), following CLSI guidelines with minor modifications. Bacterial inoculate were first prepared in phosphate-buffered saline (PBS) and adjusted to the 0.5 McFarland standard (OD_600_ ≈ 0.08–0.12), corresponding to approximately 1 × 10^8^ CFU/mL, then further diluted in PBS to obtain a working suspension of about 1 × 10^6^ CFU/mL. Two-fold serial dilutions of each extract were prepared in PBS, and 100 μL of the appropriate growth medium (Luria–Bertani broth for *S. typhimurium*, *E. coli*, and *S. aureus*; brain–heart infusion broth for L. *monocytogenes*) was dispensed into the wells of a 96-well microplate. Wells designated for test conditions received 100 μL of the extract dilution plus 2.5 μL of the standardized bacterial suspension. The positive control wells contained medium with inoculum only whereas the negative controls contained medium with extract, but no bacteria. Plates were incubated at 35 °C for 24 h, after which 20 μL of sterile resazurin solution (0.015%, *w*/*v*) was added to each relevant well and incubation continued for another 1–2 h. Wells that retained the blue color of resazurin (no change to pink) at the lowest extract concentration were recorded as the MIC, indicating complete inhibition of visible bacterial growth.

### 2.6 Preparation of dip solution

The dipping solutions were prepared from plant powders using a simple hot-water extraction. For the SF treatment, 50 g of SF powder were dispersed in 500 mL of distilled water and heated in a water bath at 60 °C for 1 h with intermittent stirring to facilitate extraction of water-soluble bioactive compounds. The mixture was then centrifuged at 4000 rpm for 15 min, and the clear supernatant was passed through Whatman No. 1 filter paper to remove residual fine particles, yielding a clarified aqueous SF extract suitable for meat dipping applications.  PAL extract was prepared in an analogous manner, using the same powder-to-water ratio and heating conditions, followed by clarification through muslin cloth filtration to retain coarse plant debris, while allowing soluble components to pass into the filtrate. The resulting SF and PAL filtrates were used directly as dipping solutions for chicken fillets in subsequent treatment and storage experiments, providing a uniform and food-grade mode of applying the plant-derived antioxidants and antimicrobials to the meat surface. This 10% (*w*/*v*) dipping solutions was selected as a practical working concentration based on the preliminary antimicrobial screening, the measurable antioxidant activity of the extracts, and should not be considered equivalent to the MIC values determined from the concentrated extract in broth microdilution.

### 2.7 Chicken fillet preparation and analysis of meat samples

Chicken breast fillets were divided into three treatment groups and immersed in the respective dipping solutions for 30 min. After treatment, excess solution was allowed to drain, and the samples were designated as: Control (dipped in distilled water only), SF-T (dipped in 10% SF extract), and PAL-T (dipped in 10% PAL extract). The fillet groups (treated and control) were then aerobically packaged in low-density polyethylene (LDPE) pouches, heat-sealed, and stored under refrigeration at 4 ± 1 °C. Quality attributes of the samples from respective groups were evaluated on days 1, 3, 5, 7, and 9.

### 2.8 Meat quality evaluation

#### 2.8.1 Proximate composition

Proximate analysis in terms of crude protein, moisture, ether extract and ash value were determined by following AOAC method (AOAC, 2011) on the day of processing of fillet samples (0 day).

#### 2.8.2 pH and water holding capacity: Technological quality dimension

The pH of control and treated (SF-T and PAL-T) chicken fillets was monitored on days 1, 3, 5, 7, and 9 of refrigerated storage using a bench-top digital pH meter equipped with a combination electrode. For each measurement, 10 g of meat were homogenized with 50 mL of distilled water for about 1 min. The slurry was allowed to stand for 5 min, gently mixed again, and the electrode was then immersed directly in the suspension to record the pH value.  The water-holding capacity (WHC) of chicken fillet was determined by a modified filter paper press method (Hamm, 1960). Approximately 500 mg of each sample was placed between folded filter papers and compressed under a 2.8 kg load for 5 min. After pressing, the meat and filter paper were weighed, and WHC was calculated as the percentage of water retained in the sample relative to its initial weight, reflecting the meat's ability to hold moisture under pressure.  

$$\mathrm{WHC}\left(\%\mathrm{retained water}\right)=100-\left(\;\frac{B-(P+\left(\mathrm{Ff}-\mathrm{Fi}\right)}{B}\;x\;100\right)$$

where, B = weight of meat sample, P = weight of pressed meat sample, Ff = final weight of filter paper after compression and Fi = initial weight of filter paper before compression.

#### 2.8.3 Thiobarbituric acid reacting substance (TBARS) values: Technological quality dimension

Lipid oxidation in chicken fillets was assessed by measuring thiobarbituric acid reactive substances (TBARS), with minor modifications (Wilson et al., 1976). In brief, 2 g of meat was homogenized with 10 mL of chilled 20% TCA for 2 min and the mixture was allowed to stand for 10 min before filtration through Whatman No. 1 filter paper.  An aliquot of 3 mL of the clear filtrate was then mixed with 3 mL of 0.1% thiobarbituric acid (TBA) solution in a test tube. The tubes were heated for 35 min at 70 °C in a water bath to develop the pink chromogen, cooled to room temperature, and the absorbance was recorded at 530 nm. TBARS values were calculated by multiplying the absorbance by a factor of 5.2 and expressed as milligrams of malondialdehyde (MDA) per kilogram of meat sample.

#### 2.8.4 Instrumental color values: Technological quality dimension

Meat color profile was assessed using a Lovibond RT-300 reflectance tintometer operating in the CIE L*a*b* color space under D65 cool white illumination and a 2° observer setting (Das et al., 2006). Finely ground meat samples were placed in petri dishes, the surface was levelled, and the sample holder was positioned flush with the instrument's measurement aperture.  From each sample, L* (lightness; 0 = black, 100 = white), a* (positive = redness, negative = greenness), and b* (positive = yellowness, negative = blueness) values were recorded at the surface. The hue angle (h°) and chroma (C*) were then calculated, wherein $h^{\circ }=\tan^{-1}\left(b^{*}/a^{*}\right)$ and $C^{*}={\left(a^{*2}+b^{*2}\right)}^{1/2}$, (respectively).

#### 2.8.5 Sensory evaluation by panelists: Sensory quality dimension

A descriptive sensory assessment of color and odor was conducted using a six-member panel, comprising faculty members, students and regular consumers of chicken products. The panelists judged color and odor using a 5-point descriptive scale adapted from Das et al. (2006), where odor scores ranged from 1 (very unpleasant) to 5 (very pleasant), and color scores from 1 (very undesirable/dull) to 5 (very desirable/bright).  The panelists received brief training regarding the attribute definitions and use of the five-point descriptive scale. Samples were coded and presented in randomized order under controlled lighting and neutral-odor conditions. Because the panel size was relatively small and the assessment was limited to color and odor, the results were interpreted as supportive descriptive sensory observations rather than as a comprehensive measure of consumer acceptability.

#### 2.8.6 Microbiological quality: Safety quality dimension

Microbiological quality of the chicken fillets was monitored on days 1, 3, 5, 7, and 9 of refrigerated storage by enumerating total coliform, *S aureus*, *E coli*, *Salmonella* spp., and total viable counts (TVC). For each treatment, 10 g of sample were aseptically transferred into a stomacher bag containing 225 mL of buffered peptone water (0.1%), homogenized for 60 s, and then serially diluted in the same diluent. From suitable dilutions, 1 mL aliquots were pour-plated with the appropriate selective media. Plate count agar was used for TVC, whereas *E. coli* and total coliforms were determined using chromocult coliform agar, after incubation at 37 °C for 48 h. In the case of *S. aureus* and *Salmonella* spp., Baird–Parker and Hektoen enteric agar were used, respectively and the plates were incubated at 37 °C for 24 h. The plates were inspected for characteristic colony morphology on each medium, and counts were expressed as log_10_ CFU per g of chicken meat.

### 2.9 Statistical analysis

All experiments were performed in triplicate and within each trial, every parameter was measured in duplicate giving a total of six observations per treatment (*n* = 6). Data from the storage study were analyzed using SPSS software (version 20.0) by two-way ANOVA to assess the effects of treatment and storage time. Treatment means were separated using Duncan's multiple range test at a 5% significance level, and results are reported as mean ± standard error (Mean ± SE).

Further, principal component analysis (PCA) was conducted on XLSTAT 2018.2 to assess the relationships and effects of different treatments on technological, sensory and safety quality dimensions. To account for variations in the scale of the response variables, a correlation-based approach was used to construct the matrix and derive the Eigenvalues. The suitability of the factorial model was assessed with the Kaiser Meyer-Olkin (KMO) test for sampling adequacy. The PCA were conducted within the three groups and also individually for each group (Control, SF-T and PAL-T).

## 3 Results and discussion

### 3.1 Antioxidants of plant extracts

#### 3.1.1 Tentative GC–MS based profiling of volatile and GC-amenable constituents

The phenolic composition of the extracts (SF and PAL), expressed as the percentage of total peak area, were determined using GC–MS analysis (Supplementary Files – Fig. S1, Table S1; Fig. S2, Table S2). In the SF extract, pyrogallol/1,2,3-benzenetriol, a polyphenolic compound, was tentatively identified as the major GC–MS -detectable constituent (37.22%). Although pyrogallic acid is essentially odourless, it can influence the overall flavor profile of foods in which it occurs (He et al., 2025). These findings align with the earlier observations of Nemkul et al. (2021).

In the PAL extract, a compound tentatively assigned as 2,6-Bis (3,4-methylenedioxyphenyl)-3,7-dioxabicyclo [3.3.0] octane (42.57%), a sesamin-related lignan, was identified as the dominant compound. Sesamin naturally occurs in sesame oil and in extracts from several plant species, including *Leucophyllum frutescens* and *Cinnamomum* spp. (Zhang, Liu, et al., 2022). This molecule exhibits notable anti-inflammatory properties and has been investigated for its potential anticarcinogenic and anticancer activities (Hadipour et al., 2023; Mottaghi & Abbaszadeh, 2022). From a sensory perspective, sesamin lacks a distinct or strong aroma but contributes to the overall phytochemical profile and may be associated with subtle scent characteristics of its source material (Yang et al., 2024).

In a previous study, bornyl acetate was reported as the principal compound in *Z. armatum* oil, followed by cymene, α-copaene, γ-terpinene, limonene, and camphene (Negi et al., 2012). The observed variations in chemical profiles may be attributed to differences in geographical conditions, cultivars, and stages of physiological development (Li et al., 2020). The drying temperature, duration, light and oxygen exposure can also alter phenolic composition and antioxidant activity; therefore, the reported profiles should be considered preparation-specific rather than absolute species fingerprints (Thamkaew et al., 2021).

#### 3.1.2 DPPH and ABTS radical scavenging activities

The DPPH and ABTS^+^ radical assays were used for evaluation of free radical-scavenging properties of the plant extracts. The results of the experiments are shown in Table 1. DPPH serves as a common free radical compound employed in assessing the free radical scavenging potential of different extracts. As DPPH undergoes scavenging and converts into DPPH-H, the solution's color shifts from purple to yellow, allowing the measurement of the degree of change through the reduction in absorbance at 517 nm (Isono et al., 2005). The lower IC_50_ value of the SF extract (31.26 μg/mL) compared with PAL (54.86 μg/mL) indicates stronger radical-scavenging potency, since a lower IC_50_ reflects a smaller quantity of extract required to achieve 50% inhibition. This suggests that the SF extract contains a higher level or more effective composition of antioxidant constituents, likely phenolics and tannins, than the PAL extract (Alsamri et al., 2021). Strong antioxidant activities of sumac fruits and *Zanthoxylum* spp. have also been reported by different researchers (Devi Heirangkhongjam & Singh Ngaseppam, 2019; Nemkul et al., 2021).

**Table 1:** Antioxidant activities of aqueous ethanolic extract of sumac fruit and prickly ash leaf.

Parameters	Sumac fruit (SF)	Prickly ash leaf (PAL)
Total phenolics content (μg GAE/mg)	130.61 ± 6.38	148.90 ± 8.8
Total flavonoid content (μg GAE/mg)	62.38 ± 2.02	21.91 ± 4.04
DPPH (IC_50_, μg/mL)	31.26 ± 0.32	54.86 ± 0.09
ABTS (IC_50_, μg/mL)	86.11 ± 5.41	100.89 ± 7.05

In ABTS^+^ assay, SF exhibited higher scavenging activity with IC_50_ value 86.11 ± 5.41 μg/mL than PAL extract, with IC_50_ value of 100.89 ± 7.05 μg/mL. Antioxidant activity of SF extract through ABTS radical scavenging activity was reported by Chhetri et al. (2020), and in *Z. acanthopodium* fruits by Witasa Dewana et al. (2022). The phenolic compounds may be responsible for the scavenging activities of the plant extracts evaluated in this study.

#### 3.1.3 Total phenolics and flavonoid contents

From the results, it was observed that the TPC of SF and PAL extracts were 130.61 ± 6.38 μg GAE/mg and 148.90 ± 8.8 μg GAE/mg, respectively (Table 1). Among the various phytochemicals present in plants, phenolic compounds are of great interest, as they can supress oxidation (lipids and proteins), inhibit the growth of spoilage and pathogenic microflora, and prevent discoloration of meat and meat products (Efenberger-Szmechtyk et al., 2020).

Flavonoids constitute the most common subgroup of phenolics in plants (Muppalla & Chawla, 2018). The flavonoid content in SF extract was found to be higher (62.38 ± 2.02 μg GAE/mg), compared to PAL extract (21.91 ± 4.04 μg GAE/mg). The presence of flavonoids in *R. chinensis* (Devi Heirangkhongjam & Singh Ngaseppam, 2019; C. Zhang et al., 2018) and *Z. acanthopodium* DC (Farida et al., 2021) have also been reported in earlier studies. However, the variation in content of phenolics and flavonoids among or within species could be due to the part of plant, extraction conditions, solvents and drying methods used (Kaewthong et al., 2019).

The contrasting antioxidant responses of SF and PAL illustrate why total phenolic concentration should not be used as the sole predictor of antioxidant performance. PAL contained approximately 14% more total phenolics than SF, yet SF exhibited substantially greater flavonoid content and lower DPPH and ABTS IC50 values. This suggests that the quality and structural characteristics of the phenolic pool, rather than its total concentration alone, were decisive in contributing to the observed radical-scavenging capacity. Phenolic hydroxyl groups are central to hydrogen-atom and electron-transfer reactions, but their antioxidant behavior is also affected by molecular substitution, conjugation, glycosylation and interactions among co-existing compounds (Charlton et al., 2023). Therefore, the stronger antioxidant response of SF may reflect a more effective combination of flavonoids and other radical-scavenging constituents.

### 3.2 Antimicrobial activities of plant extracts

#### 3.2.1 Agar well diffusion assay

The zones of inhibition (in mm) of SF and PAL extracts based on the agar well diffusion method against different foodborne bacteria is given in Fig. 1. SF extract at different concentrations (10%, 5%, 2.5%, 1.25%) exhibited *pronounced* antibacterial activity against all bacterial strains, except for *S. typhimurium* at 2.5% and 1.25%*.* The PAL extract at same tested different concentrations (10%, 5%, 2.5%, 1.25%) also showed inhibition against *S. aureus* and L. *monocytogenes*, but no activity against Gram-negative organisms.

**Fig. 1:**
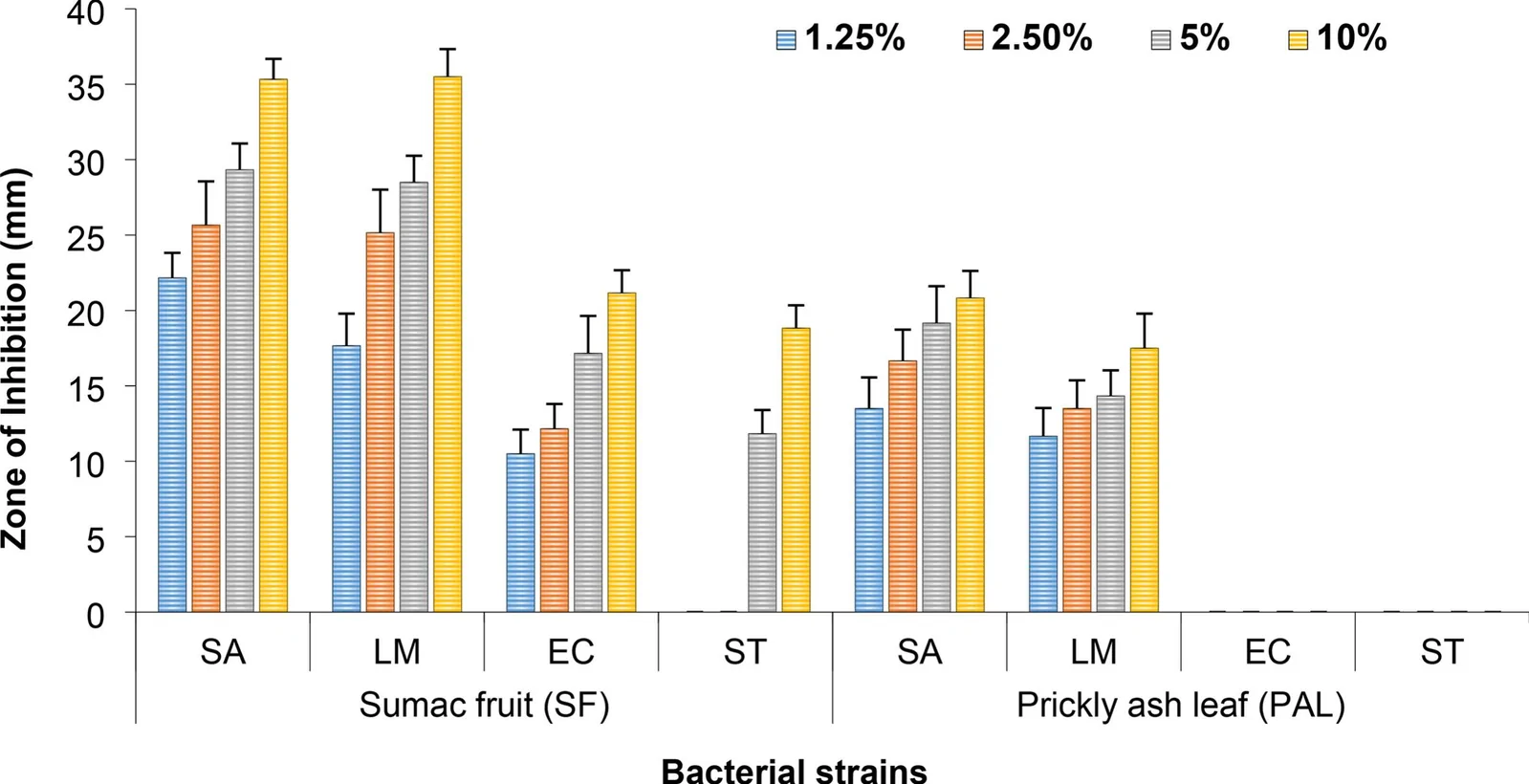
Antibacterial activity of sumac fruit (SF) and prickly ash leaf (PAL) extracts at different concentrations against the tested foodborne bacterial strains, expressed as inhibition-zone diameter (mm). Values are presented as mean ± SE. Where SA = *Staphylococcus aureus*, LM = *Listeria monocytogens*, EC = *Escherichia coli*, ST = *Salmonella Typhimurium*.

#### 3.2.2 MIC values for sumac fruit and prickly ash leaves extracts

The resazurin based micro-dilution method was used to determine minimum inhibitory concentration (MIC) values of the extracts (Supplementary file, Table S3). The MIC values for both the extracts ranged from 15 to 25 mg/mL. In a previous study, aqueous extract of sumac exhibited MIC at 50 mg/mL for *E. coli* and *S. aureus* (Poudyali & Singh, 2020)*.* Ethanol and water extracts of *Z. armatum* or PAL exhibited MIC of 40 and 20 mg/mL against *E.coli,* respectively, while aqueous extract exhibited MIC of 10 mg/mL for *S. aureus* (Negi, 2012). The antibacterial properties may be attributed to the collegial effects of several compounds, mainly phenolics present in the extracts, which inhibited various food-borne pathogens. The MIC values of 15–25 mg/mL indicate inhibitory activity under the specified broth-microdilution conditions; however, they should not be interpreted as directly equivalent to the nominal concentration of the 10% dipping solution. In meat, the effective availability of phenolic antimicrobials may be altered by interactions with muscle proteins, partitioning into lipid phases, dilution or diffusion within the meat matrix, and removal of excess treatment solution during draining. Consequently, the effective antimicrobial concentration at the meat surface may differ substantially from the nominal concentration in the dipping solution (Bouarab-Chibane et al., 2018; Oulahal & Degraeve, 2022).

### 3.3 Intrinsic quality properties of raw chicken fillets

#### 3.3.1 Technological dimension: Proximate composition

The results indicated that no significant differences (*p* > 0.05) were observed in protein, fat, or ash content between the treated (SF and PAL) and control chicken fillets (Supplementary file, Table, S4). In contrast, a significant (*p* < 0.05) reduction in moisture content was recorded in both treated groups (SF-T and PAL-T), compared to the control. The decrease in moisture content during storage is likely attributable mainly to refrigerated storage-related water loss, exudate formation and redistribution of water from the muscle matrix (Kim et al., 2020). In addition, the plant-extract coating may contribute to moisture redistribution through osmotic effects, if the solution contains sufficient soluble solids; although in thin edible coatings, this mechanism is expected to be limited. More plausibly, the coating influenced/enhanced moisture retention through surface film formation, altered water-holding capacity thereby promoting interactions between plant phenolics and muscle proteins that can reduce drip exudation (Asghari et al., 2024). Therefore, the lower moisture values observed in the treated fillets are most reasonably interpreted as the combined consequence of refrigerated-storage-related moisture loss and possible effects of the surface treatment on water distribution. Previous research on refrigerated chicken breast meat has documented progressive changes in microbial, physicochemical, and quality attributes during storage, including increased weight loss associated with moisture loss (Kaewthong et al., 2019). Again, this finding aligns with previous study demonstrating that plant-based extracts can modify moisture dynamics in meat products, without significantly affecting macronutrient composition (Zhang et al., 2016).

#### 3.3.2 Technological dimension: pH

The effect of plant extracts on pH of raw chicken meat samples studied under refrigerated storage conditions for 9 days is presented in Table 2. The ranges of pH values of control, SF-T and PAL-T during the storage study were 5.69 ± 0.02–6.42 ± 0.04, 5.63 ± 0.02–5.84 ± 0.03 and 5.67 ± 0.01–5.89 ± 0.02, respectively. In general, an increase in pH values of both control and treatments was noted during the storage study with statistically significant (*p* < 0.05) variations. It might be due to the fact that bacteria utilize amino acids liberated through protein degradation, once stored glucose is exhausted in the meat. The presence of ammonia and the products of amino acid breakdown might also have led to a rise in pH levels (Radha Krishnan et al., 2014). However, the lower pH value recorded in SF-T as compared to other groups might be due to the acidic nature of the components of SF extract solution (Raodah et al., 2014).

**Table 2:** Effect of sumac fruit (SF) and prickly ash leaf (PAL) extract on technological qualities of raw chicken fillets.

**Treatments**	**Refrigeration storage (days)**				
	**1**	**3**	**5**	**7**	**9**
**pH**					
Control	5.69 ± 0.02^eA^	5.78 ± 0.03^dA^	5.92 ± 0.02^cA^	6.17 ± 0.02^bA^	6.42 ± 0.04^aA^
SF-T	5.63 ± 0.02^dB^	5.68 ± 0.02^cdB^	5.73 ± 0.02^bcB^	5.80 ± 0.03^abB^	5.84 ± 0.03^aA^
PAL-T	5.67 ± 0.01^dA^	5.73 ± 0.01^cAB^	5.77 ± 0.02^cB^	5.84 ± 0.03^bB^	5.89 ± 0.02^aA^
**WHC**					
Control	70.26 ± 2.99	68.32 ± 2.66	65.79 ± 1.52	63.94 ± 1.22	61.63 ± 0.60
SF-T	70.61 ± 1.54	68.47 ± 1.10	67.44 ± 1.56	66.03 ± 0.66	65.14 ± 1.76
PAL-T	70.95 ± 0.98	68.76 ± 0.70	67.15 ± 1.67	66.63 ± 1.50	65.64 ± 0.71
**TBARS**					
Control	0.24 ± 0.01^dA^	0.69 ± 0.02^cA^	1.02 ± 0.05^bA^	1.13 ± 0.05^bA^	1.35 ± 0.04^aA^
SF-T	0.24 ± 0.01^eA^	0.33 ± 0.02^dB^	0.41 ± 0.02^cB^	0.47 ± 0.01^bB^	0.56 ± 0.01^aB^
PAL-T	0.23 ± 0.01^eA^	0.34 ± 0.02^dB^	0.40 ± 0.01^cB^	0.45 ± 0.02^bB^	0.61 ± 0.01^aB^

Data presented as Mean ± SE; *n* = 6; Mean values with different superscripts in each row (a - e) and column (A, B) showed significant variation (*P* < 0.05).

#### 3.3.3 Technological dimension: Water holding capacity

The water holding capacity (WHC) of treatment and control group during refrigerated storage at 4 °C for 9 d is presented in Table 2. A non-significant (*p* > 0.05) decline in WHC was found for control, SF-T and PAL-T_;_ however, the rate of decline was higher in case of control sample. It is well known that as meat spoilage progresses, it also leads to a decline in protein stability resulting in the subsequent loss of water. As the storage time samples increase, the WHC decreases, with expelling more water (Jouki & Khazaei, 2011). Although significant difference was not found between the treatments, but the values were slightly higher than the control. This might be due to the antioxidative effect of the active components which inhibited lipid oxidation and potentially helped in maintaining cell membrane stability, thereby reducing cellular moisture loss, and improving meat WHC (Kołodziej-Skalska et al., 2011). Similar findings were reported by Sadallah and Alhaji (2021), where there was a decline in WHC of lamb meat treated with tomato peel and sumac extracts during six-month storage study.

#### 3.3.4 Technological dimension: Thiobarbituric acid reacting substance (TBARS) values

Thiobarbituric acid reacting substances (TBARS) quantify secondary lipid-oxidation products, predominantly malondialdehyde (MDA) that are strongly associated with rancid off-flavors in muscle foods. Although sensory thresholds vary by matrix, a practical limit of ∼1.0 mg MDA/kg is widely used to demarcate acceptable from unacceptable oxidation in meat and meat products. In the present study (Table 2), TBARS values on day 1 did not differ among SF-T, PAL-T, and control, indicating comparable initial oxidation status. As storage progressed under refrigeration, the control exhibited significantly higher TBARS (*p* < 0.05) than both extract-treated groups. Although TBARS in SF-T and PAL-T increased gradually over 9 days, they remained within the ∼1.0 mg MDA/kg permissible range, whereas the control approached or exceeded this threshold. Such low TBARS values in SF-T sample could be due to antioxidant and radical-scavenging activities of SF extract (Bursal & Köksal, 2011). Likewise, the phenolic-rich PAL extract containing constituents such as chlorogenic acid, hyperoside, and quercitrin likely contributed to the observed attenuation of lipid oxidation in PAL-T sample, echoing findings with *Z. bungeanum* leaf extract in salted fish (Li et al., 2015) and other protein matrices. Comparable TBARS reductions have also been reported in ground sheep meat treated with sumac and barberry extracts, and in poultry treated with sumac preparations (Aliakbarlu & Mohammadi, 2015).

Notably, the preservation effects of these extracts likely arise from multiple, overlapping mechanisms rather than a single active compound. Phenolic acids and flavonoids can scavenge reactive oxygen species and lipid radicals, interrupting propagation steps in the autoxidation. They can also chelate transition metals (e.g., Fe^2+^/Fe^3+^, Cu^2+^) via hydroxyl and carbonyl groups, thereby reducing metal-catalysed initiation of lipid peroxidation in muscle (Wu et al., 2024). Together, these actions lower the formation and accumulation of secondary oxidation products measured as TBARS (Hadidi et al., 2022), as also found in treated groups of this study.

In parallel, phenolics and other extract constituents can interact with bacterial membranes, increasing permeability, disturbing proton gradients, and inhibiting essential enzymes—mechanisms that plausibly explain the concurrent reductions in microbial counts and TBARS in treated fillets (Lemoni et al., 2025). Such multifunctional activity—combining antioxidant and antimicrobial effects—is a recurring theme in studies employing plant by-product extracts to extend the shelf life of meat and meat products (Biswas et al., 2023).

#### 3.3.5 Sensory dimension: Instrumental meat color

The instrumental color values viz. lightness (L*), redness (a*), chroma (C*), and hue angle (*h°*) of raw chicken fillets treated with plant extracts (SF-T and PAL-T) and the control samples are shown in Table 3. The control samples exhibited a notable decline in lightness over time, dropping from 56.98 (day 1) to 42.70 (day 9), indicating increased darkening due to protein oxidation and microbial spoilage (Nakyinsige et al., 2015). In contrast, both SF-T and PAL-T maintained higher L* values (50.44 and 50.78, respectively, on day 9), suggesting that these extracts effectively delayed discoloration, likely due to their antioxidant properties (Zhang et al., 2016).

**Table 3:** Effect of sumac fruit (SF) and prickly ash leaf (PAL) extract on meat color values of chicken fillets.

**Treatments**	**Refrigeration storage (days)**				
	**1**	**3**	**5**	**7**	**9**
Lightness (L*)					
Control	56.98 ± 2.06^a^	54.23 ± 1.76^a^	49.72 ± 0.66^bB^	46.39 ± 0.38^bcB^	42.70 ± 0.44^cB^
SF-T	56.84 ± 0.79^a^	56.06 ± 0.81^ab^	54.89 ± 0.93^abA^	52.51 ± 1.45^bcA^	50.44 ± 1.92^cA^
PAL-T	56.56 ± 0.58^a^	55.01 ± 1.09^ab^	53.57 ± 1.05^abcA^	52.69 ± 1.56^bcA^	50.78 ± 0.87^cA^
Redness (a*)					
Control	8.65 ± 0.53^a^	8.36 ± 0.25^a^	6.99 ± 0.22^b^	4.86 ± 0.34^bB^	2.91 ± 0.25^bB^
SF-T	8.68 ± 0.39^a^	8.36 ± 0.30^ab^	7.76 ± 0.21^b^	6.86 ± 0.26^cA^	4.75 ± 0.26^dA^
PAL-T	8.70 ± 0.50^a^	8.31 ± 0.37^a^	7.73 ± 0.32^ab^	6.98 ± 0.36^bA^	5.03 ± 0.16^cA^
Yellowness (b*)					
Control	9.84 ± 0.20	10.24 ± 0.36	11.22 ± 0.23	12.30 ± 0.97	12.94 ± 0.66^B^
SF-T	9.65 ± 0.43	10.14 ± 0.49	10.52 ± 0.42	10.98 ± 0.29	11.50 ± 0.31 ^A^
PAL-T	9.72 ± 0.30	10.10 ± 0.45	10.64 ± 0.64	10.96 ± 0.46	11.18 ± 0.66 ^A^
Chroma (C*)					
Control	13.14 ± 0.31	13.24 ± 0.33	13.23 ± 0.22	13.27 ± 0.93	13.29 ± 0.60
SF-T	13.01 ± 0.41	13.15 ± 0.54	13.09 ± 0.42	12.97 ± 0.20	12.46 ± 0.27
PAL-T	13.09 ± 0.32	13.11 ± 0.41	13.16 ± 0.68	13.03 ± 0.34	12.28 ± 0.59
**Hue angle (*h°*)**					
Control	48.86 ± 2.09	50.73 ± 1.26	58.09 ± 0.98^cA^	68.06 ± 1.85^bA^	77.02 ± 1.66^aA^
SF-T	48.01 ± 1.83	50.39 ± 0.94	53.50 ± 0.93^cB^	57.96 ± 1.48^bB^	67.50 ± 1.39^aB^
PAL-T	48.30 ± 2.07^c^	50.50 ± 1.78^c^	53.87 ± 0.90^bB^	57.42 ± 2.06^bB^	65.50 ± 1.54^aB^

Data presented as Mean ± SE; *n* = 6; Mean values with different superscripts in each row (a - d) and column (A, B) showed significant variation (*P* < 0.05).

Redness (a*) decreased in all treatments over time, but the control group showed the sharpest decline (8.65 to 2.91), indicating rapid myoglobin oxidation. In contrast, SF-T and PAL-T preserved redness better (4.75 and 5.03 on day 9, respectively), likely due to polyphenolic compounds inhibiting metmyoglobin formation (Suman & Joseph, 2013). On the other hand, yellowness (b*) values were not significantly (*p* > 0.05) affected by the extract treatment during storage.

Chroma (C*) remained relatively stable across treatments, though a slight reduction was observed in both plant extract-treated samples by day 9. The hue angle (*h°*) increased, but the control had significantly higher values (77.02) compared to SF-T (67.50) and PAL-T (65.50), indicating a greater shift toward yellow-brown discoloration in untreated fillets. The greater retention of lightness and redness, together with the smaller increase in hue angle, is consistent with delayed color deterioration during storage. This may reflect reduced oxidation of myoglobin and lipids; the processes that are closely interrelated in muscle foods and contribute to discoloration and quality loss (Mancini & Hunt, 2005). However, as myoglobin redox forms, protein oxidation, extract uptake, and extract–protein interactions were not directly measured, this proposed mechanism should be interpreted cautiously. Overall, both SF and PAL extracts helped preserve the lightness and redness of chicken fillets and delayed discoloration, supporting their potential application as natural preservatives in fresh meat products.

#### 3.3.6 Sensory dimension: Sensory evaluation

The results of sensory evaluation (color and odor) of control and chicken samples treated with SF and PAL extract are given in Table 4. According to the panelists, all chicken samples exhibited good color and pleasant odor on the initial day of storage. The sensory scores of the treated samples remained higher than the control samples which started declining (*p* < 0.05) from day 5. For color attribute, there were no significant differences (*p* > 0.05) among the control, SF-T and PAL-T_,_ even though the values were slightly higher for the treatments. SF-T was paler, which might be due to application of SF extract, which is pale yellow in color due to the presence of some yellow pigments. PAL-T showed intense red color which might be due to the carotenoids present in PAL extract. Mean odor scores were significantly (*p* < 0.05) higher for the treatment samples, among which scores for samples treated with PAL extract was found to be higher. Similar enhancement of umami, color and flavor was reported in salted fish with incorporation of *Z. bungeanum* leaf extract powder (Li et al., 2015).

**Table 4:** Effect of sumac fruit (SF) and prickly ash leaf (PAL) extract on sensorial perceived quality of chicken fillets.

**Treatments**	**Refrigeration storage (days)**				
	**1**	**3**	**5**	**7**	**9**
**Color (scale 1–5)**					
Control	4.38 ± 0.02^a^	4.12 ± 0.16^ab^	3.92 ± 0.10^b^	3.37 ± 0.07^cB^	2.75 ± 0.22^dB^
SF-T	4.40 ± 0.14^a^	4.21 ± 0.15^a^	4.06 ± 0.07^a^	3.55 ± 0.14^bAB^	3.17 ± 0.22^bAB^
PAL-T	4.49 ± 0.26^a^	4.34 ± 0.18^a^	4.21 ± 0.13^a^	3.74 ± 0.03^bA^	3.33 ± 0.09^bA^
**Odor (scale 1–5)**					
Control	4.41 ± 0.09^aA^	3.81 ± 0.19^bB^	2.57 ± 0.06^cB^	1.91 ± 0.02^dB^	1.52 ± 0.02^eB^
SF-T	4.74 ± 0.04^aB^	4.45 ± 0.05^bA^	4.37 ± 0.02^bA^	3.74 ± 0.08^cA^	3.29 ± 0.03^dA^
PAL-T	4.69 ± 0.13^aAB^	4.47 ± 0.13^aA^	4.38 ± 0.10^aA^	3.84 ± 0.22^bA^	3.35 ± 0.06^cA^

Data presented as Mean ± SE; *n* = 18; Mean values with different superscripts in each row (a - d) and column (A, B) showed significant variation (*P* < 0.05).

#### 3.3.7 Safety dimension: Microbial analyses

The microbial counts such as TVC, *S. aureus*, *Salmonella* spp., *E. coli*, and total coliform counts of treated (SF-T and PAL-T) chicken fillets along with the control group was studied during refrigerated storage at 4 °C and is presented in Table 5. The initial TVC values of SF-T and PAL-T were 2.59 ± 0.18 and 3.60 ± 0.20 log_10_CFU/g, respectively which were significantly (*p* < 0.05) lower than control (4.91 ± 0.36 log_10_CFU/g). The TVC for control, SF-T and PAL-T steadily increased to 6.94 ± 0.17, 4.29 ± 0.26 and 4.65 ± 0.26 log_10_CFU/g, respectively at the end of storage. Although the TVC of SF-T and PAL-T were below the standard threshold limit of 6.7 log CFU/g till day 9 of storage, but TVC of control sample was beyond the acceptable limits on 5th day of refrigerated storage study, indicating spoilage. On comparison between treated samples, SF-T had lower TVC than PAL-T, however, the difference was minimal at the end of the preservation period. Such lower TVC in the treated groups in the first 7 days might be attributed to antimicrobial activity of sumac (Zalacain et al., 2003) and PAL extract (Muppalla & Chawla, 2018). The higher content of phenolic compounds in plant extracts is accountable for its antibacterial effect (Akagawa et al., 2003). In a similar study, lower TVC was found in fresh beef coated with 2% hydroalcoholic extract of sumac-chitosan and 4% hydroalcoholic extract of sumac-chitosan, compared to control (Langroodi et al., 2018). In another study, an extended refrigerated shelf-life of ground chicken meat packaged in active films from seed cover extract of *Z. rhetsa* was also reported, compared to control (Muppalla & Chawla, 2018).

**Table 5:** Effect of sumac fruit and prickly ash leaf extract on microbiological safety aspect of chicken fillets.

**Treatments**	**Refrigeration storage (days)**				
	**1**	**3**	**5**	**7**	**9**
**Total viable count (log**_**10**_**CFU/g)**					
Control	4.91 ± 0.36^cA^	5.68 ± 0.25^bA^	6.88 ± 0.12^aA^	6.94 ± 0.17^aA^	–
SF-T	2.59 ± 0.18^cC^	2.93 ± 0.25^bcC^	3.47 ± 0.30^abC^	3.74 ± 0.35^abB^	4.29 ± 0.26^a^
PAL-T	3.60 ± 0.20^cB^	3.87 ± 0.25^bcB^	4.16 ± 0.22^abcB^	4.36 ± 0.15^abB^	4.65 ± 0.26^a^
***Staphylococcus aureus* (log**_**10**_**CFU/g)**					
Control	1.41 ± 0.01^eA^	1.65 ± 0.03^dA^	1.90 ± 0.01^cA^	2.27 ± 0.06^bA^	2.81 ± 0.03^aA^
SF-T	1.21 ± 0.01^cC^	1.25 ± 0.01^bcC^	1.27 ± 0.01^bC^	1.36 ± 0.01^aC^	1.39 ± 0.01^aC^
PAL-T	1.32 ± 0.02^bB^	1.35 ± 0.01^bB^	1.38 ± 0.01^bB^	1.52 ± 0.02^aB^	1.57 ± 0.02^aB^
***Escherichia coli* (log**_**10**_**CFU/g)**					
Control	1.94 ± 0.02^cA^	1.98 ± 0.14^cA^	2.32 ± 0.03^bA^	2.54 ± 0.03^aA^	2.71 ± 0.02^aA^
SF-T	1.35 ± 0.02^C^	1.37 ± 0.01^B^	1.43 ± 0.01^C^	1.47 ± 0.02^C^	1.49 ± 0.14^C^
PAL-T	1.86 ± 0.02^B^	1.93 ± 0.02^A^	1.95 ± 0.04^B^	1.96 ± 0.06^B^	1.98 ± 0.11^B^
**Total coliform count (log**_**10**_**CFU/g)**					
Control	2.84 ± 0.18^cA^	3.14 ± 0.10^bcA^	3.36 ± 0.03^bA^	3.57 ± 0.26^abA^	3.91 ± 0.07^aA^
SF-T	1.42 ± 0.04^B^	1.67 ± 0.10^B^	1.71 ± 0.19^B^	1.75 ± 0.21^C^	1.83 ± 0.13^C^
PAL-T	1.72 ± 0.02^bB^	1.76 ± 0.04^bC^	1.81 ± 0.01^bB^	2.61 ± 0.03^aB^	2.80 ± 0.17^aB^
***Salmonella* spp. (log**_**10**_**CFU/g)**					
Control	ND	ND	ND	ND	ND
SF-T	ND	ND	ND	ND	ND
PAL-T	ND	ND	ND	ND	ND

Data presented as Mean ± SE; *n* = 6; Mean values with different superscripts in each row (a - e) and column (A, B, C) showed significant variation (*P* < 0.05); ND: Not detected.

The *S. aureus* count of control was significantly higher (*p* < 0.05) than the treated fillet samples_._ Although, the count gradually increased (*p* < 0.05) for all samples, it was slow in case of SF-T and PAL-T. This might be accounted to antimicrobial activity of different solvent extracts of *R chinensis* Mill against *S. aureus,* as also reported by some researchers (Poudyali & Singh, 2020). Similarly, varying degree of antibacterial activity of *Z. acanthopodium* against Gram-positive was reported where the highest activity was noted against *S. aureus* (Majumder et al., 2014). In another study, minced fish fillets inoculated with *S. aureus* were treated by immersion in *R. coriaria* fruits extract and it was found that the count was reduced in the surface and inner parts of samples (Tayel et al., 2021). Similar results were also observed in chicken meat packed in active films with *Z. rhetsa* extract (Muppalla & Chawla, 2018).

The *E. coli* count of control sample was significantly higher (*p* < 0.05) than the treatments_._ Lower count observed in treated samples can be linked to antibacterial effects of *R. chinensis* Mill against *E. coli* (Poudyali & Singh, 2020) and *Z. acanthopodium* against *E. coli* (Majumder et al., 2014). Although there was a significant (*p* < 0.05) and gradual increase in total coliform count of control and PAL-T, the increase was non-significant (*p* > 0.05) for SF-T. This could be due to inhibitory effects of SF on coliform, as also reported by (Aliakbarlu & Mohammadi, 2015).

*Salmonella* spp. was not detected in control as well as chicken fillets treated with SF and PAL extract during the 9 d refrigerated storage study. Absence of *Salmonella* in the meat samples might be due to good handling procedures in preparation of meat samples and hygienic laboratory conditions (Langroodi et al., 2018).

Nevertheless, the antimicrobial behavior of the extracts appeared to vary according to both the botanical matrix and the target organism. SF demonstrated activity against both Gram-positive and Gram-negative bacteria, whereas PAL showed a more pronounced inhibitory response against Gram-positive organisms, particularly in relation to *S. aureus*. Such differences may be attributed to variations in bacterial cell-envelope architecture, which influenced the accessibility and efficacy of plant-derived antimicrobial constituents.

Nonetheless, the antimicrobial effects as observed in the present study should not be interpreted as evidence of a single, specific mechanism. Since the study measured microbial growth inhibition rather than membrane permeability, intracellular leakage, or interactions with defined molecular targets, the precise mode of action cannot be established (Hossain, 2024). It is therefore more appropriate to consider the observed inhibition as the combined effect of multiple constituents present in the extracts, potentially acting synergistically through overlapping mechanisms, including interactions with bacterial cell-envelope components, alteration of membrane permeability, interference with intracellular processes, and disruption of cellular homeostasis (Vaou et al., 2021). Overall, the lower microbial counts and delayed increase in TVC, *S. aureus*, *E. coli*, and total coliforms in the treated samples demonstrate the potential of SF and PAL extracts to improve the microbiological safety and refrigerated storage stability of the chicken fillets.

### 3.4 Multidimensional analyses: Effects of treatments and storage time on intrinsic meat quality properties

The principal component analysis (PCA) was employed to comprehensively evaluate the multidimensional relationships among the variables and treatments, and also to evaluate the evolution of the three intrinsic quality dimensions (safety, technological, and sensory) in chicken fillets treated with SF and PAL extracts during refrigerated storage. The PCA (Fig. 2, Fig. 3) revealed distinct clustering patterns, highlighting the differential effects of treatments and storage time as a consequence of dynamic changes in the meat quality traits assessed.

**Fig. 2:**
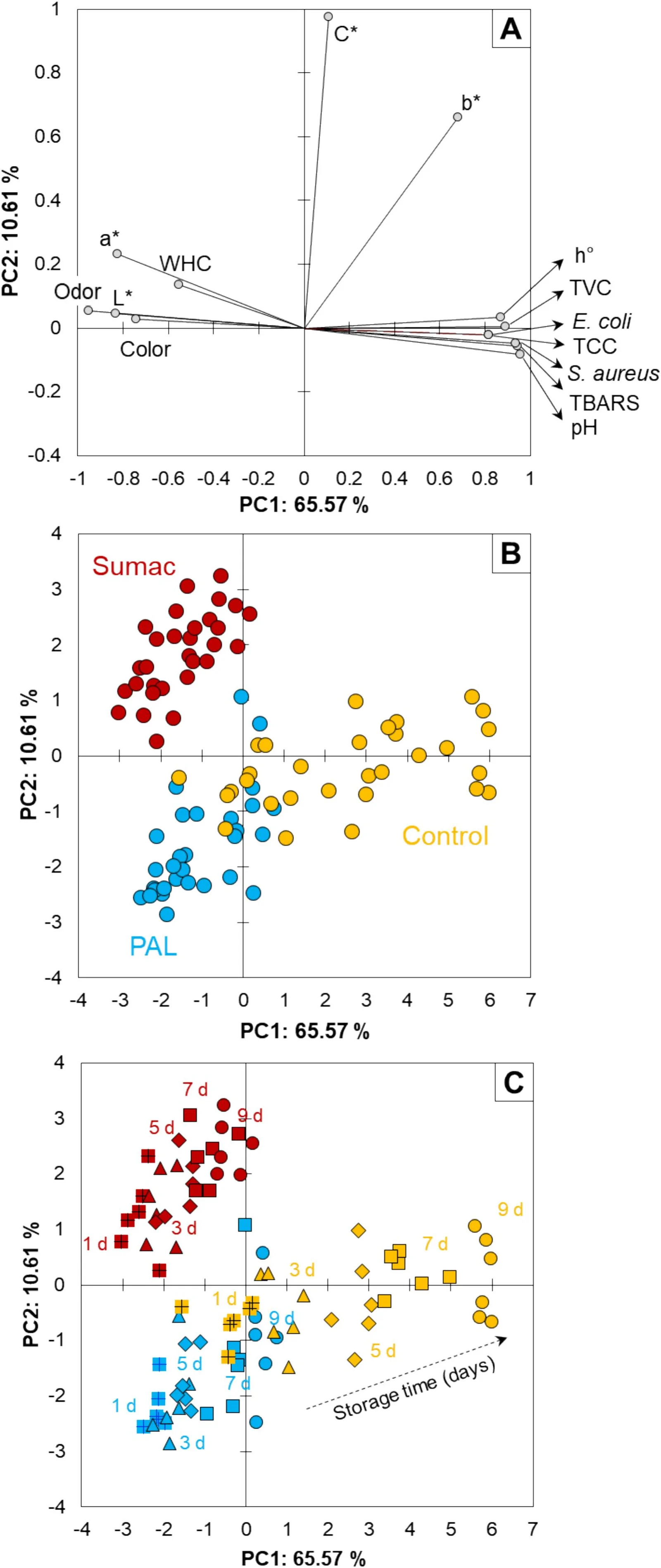
Principal Component Analysis (PCA) of chicken fillet quality traits within the three sample groups during refrigerated storage. **A)** Projection of the meat quality traits variables within the two first principal components. **B)** Bi-plot highlighting the projection of the observations for the control, PAL and SF groups. **C)** Bi-plot within the same control, PAL and SF groups highlighting the projection of the observations over storage.

**Fig. 3:**
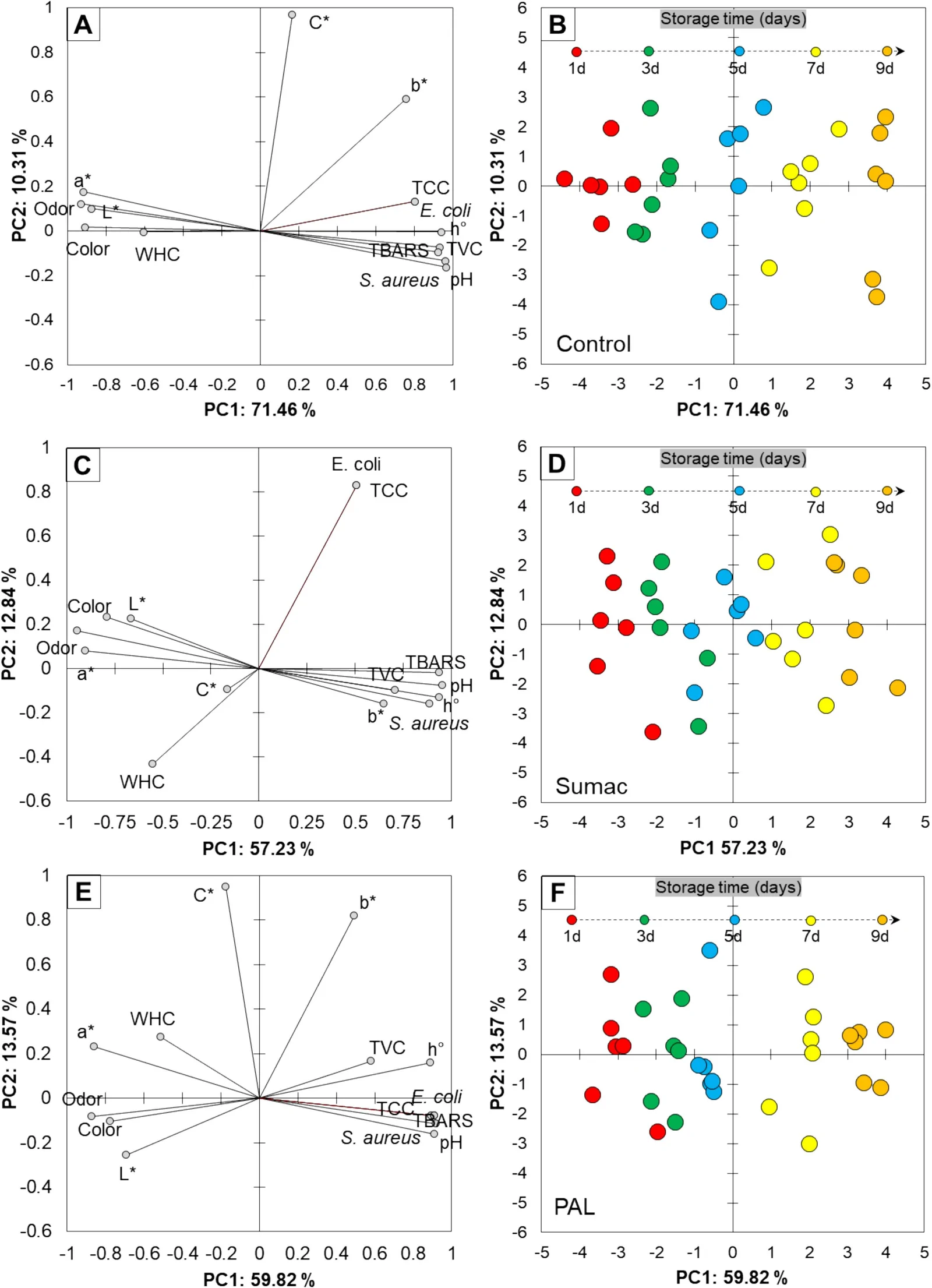
Principal Component Analysis (PCA) of chicken fillet quality traits during refrigerated storage depicting for each group the projection of the variables and the related bi-plot for the control (A, B); Sumac fruit (C, D), and PAL extract (E, F). the bi-plot further depicts the temporal dynamics from days 1 to 9.

The two principal components (PC1 and PC2) in Fig. 2 explained 76.18% of the total variance, with positive side of PC1 primarily associated with oxidative stability (TBARS), pH, h° and microbial load (TVC, TCC and all microorganisms assessed), while the negative side is correlated with sensory attributes (color, odor), technological properties (WHC) and color (L* and a*). The PC2 that is not contributing to significant separation, has C* and b* in the top side and drive the sumac group into the top.

The PCA clearly separated the treatments into distinct clusters (Fig. 2), where control samples grouped with higher TBARS, microbial counts, and hue angle (h°), indicating rapid spoilage and oxidative degradation. On the other hand, SF treated samples correlated strongly with antioxidant activity (lower TBARS) and retained redness (a*), attributed to sumac's high flavonoid content (62.38 μg GAE/mg) and DPPH scavenging capacity (IC_50_: 31.26) (Table 1). This aligns with findings by Bursal & Köksal (2011), where sumac extracts mitigated lipid oxidation. Again, PAL-treated samples associated with improved sensory scores (odor, color) due to carotenoids and lignans in PAL, corroborating earlier reports that used *Zanthoxylum* extracts to enhance meat color stability (Li et al., 2015).

Furthermore, temporal shifts in the observations of the storage times in the PCA trajectories (Fig. 2) revealed that during day 1–5, control samples diverged sharply from treated groups, driven by rising TBARS and microbial counts. But during day 7–9, both SF and PAL maintained proximity to freshness markers (high L*, a*), while control samples shifted toward spoilage-associated variables (high h°, TVC). This mirrors to an earlier study where untreated meat exhibited rapid quality deterioration (Nakyinsige et al., 2015).This further aligns with previous studies, where PCA successfully discriminated meat quality traits based on oxidative and microbial spoilage markers (Das et al., 2020; Zhang et al., 2016). Notably, TBARS and redness (a*) were inversely correlated, reflecting the role of lipid oxidation in meat discoloration (Mancini & Hunt, 2005). Microbial counts (TVC) clustered closely with storage time, emphasizing their progressive increase whatever the groups (Fig. 3), but more importantly in the control samples (Fig. 3A and B).

The multidimensional analysis further underscored the preservation effects of SF and PAL extracts, where the former dominated in antioxidant and antimicrobial efficacy (lower TBARS, TVC), linked to its phenolic acids (e.g., pyrogallic acid, 37.22%) (Supplementary file, Table S1). PAL extract excelled in sensory preservation, likely due to lignans thereby stabilizing color and odor (Farida et al., 2021). The PCA validated that SF and PAL extracts differentially preserve chicken fillets: the former extends shelf-life via oxidative and microbial inhibition, while the later enhances sensory appeal. This multidimensional approach aligns with global trends in natural preservative research (Efenberger-Szmechtyk et al., 2020), demonstrating the potential of plant extracts to replace synthetic additives in meat products.

## 4 Conclusion

Overall, the present study demonstrates that sumac fruit (SF) and prickly ash leaf (PAL) extracts can differentially modulate the quality attributes of raw chicken fillets during refrigerated storage. The stronger radical-scavenging activity of the SF extract was accompanied by greater control of lipid oxidation and microbial growth in chicken fillets, whereas the PAL extract showed comparatively favorable sensory responses. These differences were consistent with the distinct phytochemical profiles of the two botanical materials and were further visualized by multivariate analysis. The findings support the potential of these extracts as natural preservation ingredients for refrigerated poultry; however, the present results should be interpreted as evidence of quality-preservation potential rather than definitive proof of pathogen inactivation or molecular mechanisms. Further work involving targeted quantification of individual bioactive compounds, characterization of extract–meat interactions, coupled with validation using larger sensory panels would help establish the mechanistic and technological potential of these botanical extracts more conclusively.

## CRediT authorship contribution statement

**Sushmita Moirangthem:** Writing – original draft, Methodology, Investigation, Formal analysis. **Gopal Patra:** Writing – review & editing, Supervision, Methodology. **Subhasish Biswas:** Writing – review & editing, Resources, Methodology. **Laura Alessandroni:** Writing – review & editing, Software, Methodology. **Santanu Nath:** Validation, Methodology, Investigation. **Arun K. Verma:** Validation, Methodology, Investigation. **Pramod Kumar Nanda:** Writing – review & editing, Writing – original draft, Resources, Methodology. **Arun K. Das:** Writing – review & editing, Writing – original draft, Visualization, Validation, Supervision, Resources, Methodology, Funding acquisition, Formal analysis, Data curation, Conceptualization. **Mohammed Gagaoua:** Writing – review & editing, Visualization, Supervision, Software, Methodology, Funding acquisition, Formal analysis, Data curation, Conceptualization.

## Declaration of competing interest

The authors declare that they have no known competing financial interests or personal relationships that could have appeared to influence the work reported in this paper.

## Data Availability

The raw data supporting the findings of this research are available from the corresponding author upon reasonable request.
