# Supplementary material for: Effects of sumac fruit and prickly ash leaf extract on the intrinsic technological, sensory and safety quality properties of chicken fillets
Source: Food Chem X. 2026 Oct 26;40:104691. doi: 10.1016/j.fochx.2026.104691 (PMC13770599; doi:10.1016/j.fochx.2026.104691)
Supplement: Supplementary file 1 [file mmc1.docx]

**Figure S1*.*** GC-MS Total Ion Chromatogram (TIC) of sumac fruit extract

**Table S1. GC–MS-detectable constituents tentatively identified in sumac fruit extract**

| **Peak** | **Retention Time** | **Area%** | **Tentative compound identification** |
| --- | --- | --- | --- |
| 1 | 3.405 | 2.62 | 2,5-Furandione, dihydro-3-methylene |
| 2 | 4.147 | 1.45 | Oxiranemethanol |
| 3 | 6.728 | 1.15 | 5-Hydroxymethylfurfural |
| 4 | 10.892 | 37.22 | 1,2,3-Benzenetriol |
| 5 | 30.844 | 21.68 | Pentadecanoic acid |
| 6 | 36.030 | 13.86 | 9,12-Octadecadienoic acid, methyl ester |
| 7 | 36.192 | 10.91 | cis-9-Hexadecenal |
| 8 | 36.300 | 1.48 | cis-Vaccenic acid |
| 9 | 36.552 | 0.93 | cis-Vaccenic acid |
| 10 | 36.866 | 3.46 | Octadecanoic acid |
| 11 | 45.608 | 0.79 | Rotundene |
| 12 | 50.264 | 0.95 | Bi-1-cycloocten-1-yl |
| 13 | 50.855 | 2.18 | Rotundene |
| 14 | 53.658 | 1.31 | 2,4-Di-tert-butyl-6-(tert-butylamino) phenol |

**Figure S2.** GC-MS Total Ion Chromatogram (TIC) of prickly ash leaf extract

**Table S2.** **GC–MS-detectable constituents tentatively identified in prickly ash leaf extract**

| **Peak** | **Retention Time** | **Area %** | **Tentative compound identification** |
| --- | --- | --- | --- |
| 1 | 3.494 | 0.59 | Eucalyptol |
| 2 | 30.744 | 1.25 | Pentadecanoic acid |
| 3 | 60.927 | 2.39 | 2,6-Bis (3,4-methylenedioxyphenyl)-3,7-dioxabicyclo [3.3.0] octane |
| 4 | 61.744 | 1.66 | 2,6-Bis (3,4-methylenedioxyphenyl)-3,7-dioxabicyclo [3.3.0] octane |
| 5 | 62.030 | 7.06 | 3-Benzofuranmethanol, 2,3-dihydro-2-(4-hydroxy-3-methoxyphenyl)-5-[3-hydroxy-1-propenyl]-7-methox |
| 6 | 62.143 | 42.57 | 2,6-Bis (3,4-methylenedioxyphenyl)-3,7-dioxabicyclo [3.3.0] octane |
| 7 | 62.924 | 2.78 | 1H,3H-Furo [3,4-c] furan, 1,4-bis (3,4-dimethoxyphenyl) tetrahydro-, [1R-(1.alpha.,3a.alpha.,4.alpha.,6a.alp |
| 8 | 63.200 | 41.09 | 1H,3H-Furo[3,4-c] furan, 1,4-bis (3,4-dimethoxyphenyl) tetrahydro-, [1R-(1.alpha.,3a.alpha.,4.alpha.,6a.alp |

**Table S3.** MIC values for sumac fruit and prickly ash leaf extract

| **Bacterial strains** | **MIC (mg/mL)** | |
| --- | --- | --- |
|  | **Sumac fruit** | **Prickly ash leaf** |
| *Staphylococcus aureus* | 25 | 25 |
| *Listeria monocytogenes* | 25 | 25 |
| *Escherichia coli* | 15 | 15 |
| *Salmonella* Typhimurium | 15 | 25 |

**Table S4.** Proximate composition of chicken fillets and their comparison within the three groups

| **Treatments** | **Moisture %** | **Protein %** | **Fat %** | **Ash %** |
| --- | --- | --- | --- | --- |
| Control | 73.77±0.73^A^ | 21.55±0.43 | 2.52±0.14 | 1.45±0.28 |
| SF-T | 71.74±0.01^B^ | 21.57±0.33 | 2.51±0.10 | 1.44±0.23 |
| PAL-T | 71.66±0.01^B^ | 21.56±0.33 | 2.55±0.02 | 1.46±0.18 |

Data presented as Mean ± SE; n = 6; Mean values with different superscripts in each column showed significant variation (*P* < 0.05).
